# Dr Jim Birley

**DOI:** 10.1192/pb.bp.114.046987

**Published:** 2014-04

**Authors:** Peter Tyrer, Robert van Voren

**Figure:**
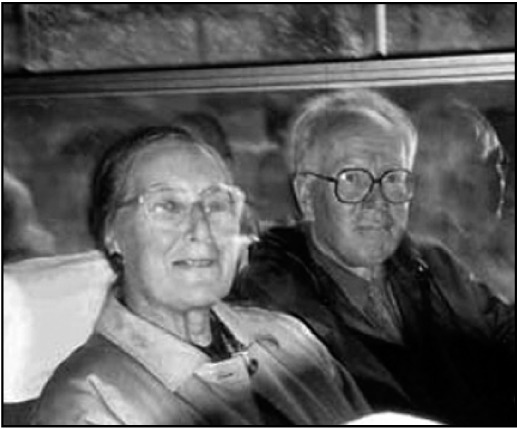
Jim and Julia Birley at one of the meetings of the Network of Reformers in Psychiatry, Czech Republic, 1995.

## Formerly Consultant Psychiatrist at the Maudsley Hospital and President of the Royal College of Psychiatrists (1987-1990)

Forty years ago a group of psychiatrists training at the Maudsley Hospital were discussing the attributes of a good psychiatrist. They came to some sort of soggy consensus, but then decided to personalise it by asking each member to note down which psychiatrist they would choose to be referred to if they became mentally ill. There were over 50 to choose from but 77% of them voted for the same person, Jim Birley. So, even this relatively junior group recognised there was something particularly special about this unassuming man who shone forth more brightly than his many more flamboyant glitterati at the Bethlem and Maudsley hospitals. In the ensuing years, both authors of this obituary experienced ample reinforcement of the reasons why Jim was particularly special.

He came from a distinguished family, with his father, also Dr James Birley, famed for his original work on fatigue in pilots in the First World War, as a model to follow. Jim moved effortlessly from Winchester, where he was head boy, to University College, Oxford, before entering psychiatry, where he passed through the memorable, if occasionally disturbing, hands of William Sargant at St Thomas’ Hospital before going on to the Maudsley Hospital in 1960, where he stayed for the rest of his working life. But at the Maudsley he took a different course from many of his contemporaries. He was training to be a social psychiatrist, and good social psychiatrists needed to know their patients, so he became drawn towards the many problems of the patients in Camberwell.

Our impression at the time, perhaps influenced too heavily by juvenile cynicism, was that most of Jim’s colleagues regarded Camberwell folk as fodder for their personal advancement at the foremost psychiatric centre of excellence, and the trials and troubles of local residents were not of nearly as much interest as their psychopathology. But Jim never thought of personal advancement in this way. As a budding social psychiatrist he needed to understand all their problems in context, and he was the first consultant at the Maudsley Hospital to argue the case for a catchment area for the hospital, founding the Southwark Association for Mental Health, and putting great energy into developing a day centre and a housing association to provide the full range of social care.

What was amazing is that he managed to combine this coalface psychiatry, an enterprise supported with generous enthusiasm by his wife Julia, with both academic and administrative responsibilities. In 1965 he became a member of the Medical Research Council Social Psychiatry Unit at the Institute of Psychiatry, joining George Brown to work on social influences on psychotic illness. Their paper^1^ was one of the first to show the link between life events and schizophrenia. Jim then became Dean of the Institute of Psychiatry between 1971 and 1982. This was a time when the Institute was expanding greatly, not without conflict, and during this time Jim had to deal with an aggressive campaign attempting to close it down. In responding to this threat he developed an unequivocal manic episode, no doubt a personal confirmatory evidence of the association between life events and psychosis. Characteristically, and with an openness that was very unusual at the time, he did not mind talking to others about this episode, and indeed commented amusingly, ‘It was very pleasant, and I would have liked it if it gone on for a little longer’. This event did not prevent him becoming Dean of the Royal College of Psychiatrists between 1982 and 1987, and subsequently President of the College in 1987, and later President of the British Medical Association for 1993-1994. He was awarded the CBE in 1990.

Despite this accession to the highest offices of psychiatry in the land, Jim never lost his enthusiasm for what is now called, rather clumsily, person-centred psychiatry. It is an aspiration of many but achieved genuinely by very few. He had an abiding passion for fairness and equity in all parts of mental health practice, and this continued in his international role in helping to expose the political abuses of psychiatry. He was Chair of the Geneva (subsequently Global) Initiative on Psychiatry (GIP) for most of the 1990s, and one of his major successes was to persuade the Soviet Foreign Ministry at the World Psychiatric Association meeting in Athens in 1989 to acknowledge the existence of systematic political abuse of psychiatry in their country. Following this, Soviet psychiatry returned to membership of the Association. Jim subsequently became an active participant in the Network of Reformers in Psychiatry that GIP established in 1994, and which during the next 10 years formed the main engine for mental health reform in Central and Eastern Europe and the former USSR. Over the years he developed many close friendships in the region, generously offering advice and support, yet never failing to give his opinion, however unpleasant it might be. We can remember many occasions when his anger about inertia, blinkered vision or outright abuse was not only visible but also quite audible, but the origin of his anger was always his continuing support for persons with mental illness and their rights.

This abiding passion for fairness extended to colleagues also. When Ellen Mercer, Director of International Affairs at the American Psychiatric Association (APA), was told to clear her desk in 1999 after almost 25 years of service, Jim was so angry that he returned his medal as Distinguished Fellow of the APA with a letter, in which he wrote that ‘an organisation capable of dumping a person just a few months away from the 25th anniversary of employment with the organisation in the rudest possible manner and without even offering the chance to negotiate a fair and generous compensation, is clearly an organisation that no longer values human beings and human dignity - and is certainly not the organisation that played such a prominent role in the fight against the political abuse of psychiatry’.

Even when he retired altogether 3 years ago because of the progression of Alzheimer’s disease, he could still be roused to protest at perceived inequities and unfairness: ‘I’ve got Alzheimer’s you know, but some things still get through and I can still get angry’. And so he was, to the end, a fighter for the rights and values of the disadvantaged, whether caused by mental illness, political abuse or just plain unfairness, and we are blessed that he never lost his dominant place on the higher ground.

Jim Birley loved and lived in Herefordshire. He is survived by his wife, Julia, their son and three daughters.

Dr Jim Birley, born 31 May 1928, died 6 October 2013.
